# Deletion of ΔdblGata motif leads to increased predisposition and severity of IgE-mediated food-induced anaphylaxis response

**DOI:** 10.1371/journal.pone.0219375

**Published:** 2019-08-01

**Authors:** Sribava Sharma, Sunil Tomar, Mayuri Dharne, Varsha Ganesan, Andrew Smith, Yanfen Yang, Lisa Waggoner, Yui-Hsi Wang, Simon P. Hogan

**Affiliations:** 1 Division of Allergy and Immunology, Department of Pediatrics, University of Cincinnati College of Medicine, Cincinnati Children’s Hospital Medical Center, Cincinnati, OH, United States of America; 2 Immunobiology graduate program, Division of Immunobiology, Department of Pediatrics, University of Cincinnati College of Medicine, Cincinnati Children’s Hospital Medical Center, Cincinnati, OH, United States of America; 3 Mary H Weiser Food Allergy Center, Department of Pathology, Michigan Medicine, University of Michigan, Ann Arbor, MI, United States of America; Université Paris Descartes, FRANCE

## Abstract

**Background:**

Previous studies have revealed an important role for the transcription factor GATA-1 in mast cell maturation and degranulation. However, there have been conflicting reports with respect to the requirement of GATA-1 function in mast cell dependent inflammatory processes. Herein, we examine the requirement of GATA-1 signaling in mast cell effector function and IgE-mast cell-dependent anaphylaxis.

**Objective:**

To study the requirement of GATA-1 dependent signaling in the development and severity of IgE-mast cell-dependent anaphylaxis in mice.

**Methods:**

Wild type (Balb/c) and mutant ΔdblGata (Balb/c) mice were employed to study the role of GATA-1 signaling in *in vitro* IgE-mediated activation of bone marrow derived mast cells (BMMCs). Murine models of passive IgE-mediated and oral antigen-induced IgE-mediated anaphylaxis were employed in mice. Frequency of steady state mast cells in various tissues (duodenum, ear, and tongue), peritoneal cavity, and clinical symptoms (diarrhea, shock, and mast cell activation) and intestinal Type 2 immune cell analysis including CD4^+^ Th_2_ cells, type 2 innate lymphoid cells (ILC2), and IL-9 secreting mucosal mast cells (MMC9) were assessed

**Results:**

In vitro analysis revealed that ΔdblGata BMMCs exhibit a reduced maturation rate, decreased expression of FcεRIα, and degranulation capacity when compared to their wildtype (WT) counterparts. These *in vitro* differences did not impact tissue resident mast cell numbers, total IgE, and susceptibility to or severity of IgE-mediated passive anaphylaxis. Surprisingly, ΔdblGata mice were more susceptible to IgE-mast cell-mediated oral antigen induced anaphylaxis. The increased allergic response was associated with increased Type 2 immunity (antigen-specific IgE, and CD4^+^ T_H_2 cells), MMC9 cells and small intestine (SI) mast cell load.

**Conclusion:**

Diminished GATA-1 activity results in reduced in vitro mast cell FcεRIα expression, proliferation, and degranulation activity. However, in vivo, diminished GATA-1 activity results in normal homeostatic tissue mast cell levels and increased antigen-induced CD4^+^ Th2 and iMMC9 cell levels and heightened IgE-mast cell mediated reactions.

## Introduction

Anaphylactic reactions are characterized by Type I Hypersensitivity responses mediated by IgE-mast cell-dependent processes [1–4]. IgE, produced in response to foreign antigens, crosslinks FcεRIα on mast cells triggering degranulation and release of mast cell mediators such as tryptase, histamine, and leukotrienes which are thought to drive both local and systemic symptoms [1, 2, 5]. The most common IgE-mast cell dependent disease is food allergy [6, 7]. It is estimated to effect 6 million children, and 9 million adults in the U.S., costing nearly $25 billion per year [8, 9]. The involvement of mast cells in IgE-mediated anaphylaxis has been established with clinical and experimental evidence [10–14]. Clinically, elevated, levels of antigen specific IgE and primary mast cell mediators, such as tryptase and histamine are elevated in patients experiencing allergic reactions [10–12]. Consistently with this, studies involving FcεRI knockout mice and mast cell deficient mice have demonstrated a requirement for IgE-FcεRI-mast cell axis in the onset of symptoms of IgE-mediated responses [13, 14].

Mast cells are derived from bone marrow hematopoietic stem cells which is a process regulated by various transcription factors including PU.1, MTIF, STAT5, C/EPBα, and GATA-2 [15–19]. GATA-1, a zinc-finger protein, is expressed in a number of hematopoietic lineages such as mast cells, eosinophils, megakaryocytes, and erythroid cells [20, 21]. Sertoli cells are the only non-hematopoietic cells that express GATA-1 [22]. Loss of function studies have revealed an important role for GATA-1 in the development of megakaryocytes and erythroids [23–25]. Indeed, genetic deletion of GATA-1 results in the pre-term death of mice due to anemia and improper megakaryocyte development [23–25]. To study GATA-1 biology, investigators have generated a number of mice lines with reduced GATA-1 expression [26–29]. Notably, studies utilizing these mice have revealed somewhat conflicting roles for GATA-1 in mast cell development and function [26–29]. Studies utilizing GATA-1^low^ mice revealed that decrease of GATA-1 expression results in reduced mast cell FcεRIα expression and altered immature mast cell numbers [26]. In contrast, a study employing conditional GATA-1 knockout mice (Gata1^−/y^) showed that deletion of GATA-1 in adult mice had little to no effect on tissue resident mast cells or BMMCs [27]. Finally, using the ΔdblGata mice, where the palindromic double GATA site upstream of the GATA-1 promoter has been genetically deleted [28, 30, 31], investigators revealed normal mast cell levels in the context of airway inflammation [29]. However, the requirement of GATA-1 signaling for IgE-mast cell dependent responses has not been investigated. In this study, we employ ΔdblGata mice to investigate the role of GATA-1 signaling in mast cell development and IgE-mast cell dependent anaphylaxis reactions. We show that GATA-1 signaling is necessary for normal IgE-mediated bone-marrow derived mast cell degranulation and maturation, however diminished GATA-1 signaling did not impact *in vivo* mast cell maturation and IgE-MC-dependent responses. In fact, ΔdblGata mice exhibit increased antigen-induced CD4^+^ Th2 and ILC2 response and a more severe IgE-mediated reaction suggesting that diminished GATA-1 signaling augments IgE-mast cell mediated reactions in vivo.

## Material and methods

### Animals

ΔdblGata (BALB/C) mice were generously provided by Dr. Stuart H. Orkin (Harvard, MA, USA) [28] and maintained on wild-type (WT) BALB/C mice originally provided by Charles River Laboratories, (Wilmington, MA, USA). Age-, sex-, and weight-matched littermates were used in all experiments. The mice were maintained and bred in a clean barrier facility at Cincinnati Children’s Hospital Medical Center (CCHMC) and University of Michigan and were handled under an approved Institutional Animal Care and Use Committee protocol. All experimental procedures were approved by the Institutional Animal Care and Use Committee of Cincinnati Children’s Medical Center and University of Michigan.

### IgE-mediated experimental food allergy

For the skin sensitization food allergy model, mice were first sensitized by applying (painted on skin surface) 20 μl of MC903 (0.1 μM Calcipotriol, TOCRIS Bioscience) and 5 μl of OVA (200mg/ml) to the right ear for 14 days consecutively. After the sensitization phase, the mice were fasted for 4 hours and then orally gavaged with OVA (50 mg in 250 μl saline) eight times every other day. The mice were observed for evidence of allergic symptoms 60 minutes following challenge as previously described [32]. In experiments to test for requirement of IgE, food allergic BALB/c mice received a single injection of either isotype control (rat IgG2a mAb; clone bGL117; i.p. 5μg / 200μl) or rat anti-mouse IgE (rat IgG2a mAb; clone EM95; i.p. 5μg / 200μl) twenty-four hours prior to o.g. challenge eight. Administration of 5μg dose of the activating rat IgG2a mAb to mouse IgE, EM-95, has been shown to induce anaphylaxis characterized by decreased mobility and hypothermia[33] and subsequently desensitize mice to IgE-mediated responses for seventy two hours[34]. Twenty-four hours later mice received oral gavage challenge and evidence of allergic symptoms 60 minutes following challenge was examined. For the i.p. sensitization food allergy model, mice were sensitized with 50 μg of OVA and 1 mg of alum in sterile saline by intraperitoneal (i.p.) injection. Beginning 2 weeks later, mice were held in the supine position three times a week for 3 weeks and orally gavaged with 250 μL of OVA (50 mg) in saline or 250 μL of saline [vehicle (Veh)]. Before each i.g. challenge, mice were deprived of food for 3 to 4 hours. Challenges were performed with i.g. feeding needles (01-290-2B; Fisher Scientific Co.). Rectal temperatures were measured before and 60 minutes after OVA challenge. Diarrhea was assessed by visually monitoring mice for up to 60 minutes after i.g. challenge. Mice showing liquid stool were recorded as diarrhea-positive.

### Clinical measurement parameters of food allergy

Mice were scored using a scoring system after the eighth challenge [35]. 0 for no clinical symptoms, 1 for repetitive nose and ear scratching, 2 for lethargy, pilar erecti and puffy nose, ears, and mouth. 3 for periods of motionless for >1 min and lying prone. 4 for no response to whisker stimuli or prodding. 5 for tremor, convulsions, and death. Occurrence of diarrhea, in the form of liquid excretion post challenge, was tracked within 1 hour after every challenge. For the purpose of Clinical measurement of food allergy, the investigator was blinded to the identity of the respective treatment groups.

### ELISA and histological measurements

Serum samples from blood drawn after cardiac puncture were analyzed using ELISA kits for OVA-specific IgE (MD Bioproducts, Oakdale, MN, USA), MCPt-1 (Invitrogen, Carlsbad, CA, USA), and OVA-specific IgG1 (Alpha Diagnostic International, San Antonio, TX, USA). Total IgE (Bioscience, San Diego, CA, USA) was performed for steady state analysis. For intestinal histological analyses, duodenal tissue was fixed in 10% formalin and processed by standard histological procedures as previously described [36]. At least eight random images were taken from at least three random sections per mouse. Quantification of stained cells per square millimeter was performed by morphometric analysis using Image Processing Software (ImagePro, Media Cybernetics, MD, USA).

### Lamina propria mononuclear isolation

Mice were euthanized in CO_2_ and SI were removed surgically from mice, cut longitudinally, and cleaned in HBSS as previously described [37]. In brief, samples were incubated in HBSS with 5mM EDTA (HBSS-EDTA) at 4°C for 5 minutes followed by vortexing in fresh HBSS-EDTA for four cycles to remove epithelial cells. The remaining tissues were minced in 8 mL RMPI 1640 (Gibco, Grand Island, NY, USA) with 2.4mg/mL collagenase A (Roche Basel, Switzerland) and 0.2mg/mL DNase I (Roche, Basel, Switzerland) at 37°C for 30 minutes. Single cell suspensions were obtained by passing the digested tissue 4 times through a 10mL syringe using a 19G needle and filtering homogenate. Filtered cells were washed once with RPMI 1640, suspended in 5mL of 44% Percoll and underlayed with 3mL of 67% Percoll before centrifugation for 20 minutes at room temperature (24°C) at reduced acceleration and deceleration. Lamina propria cells were collected from the interface between 44% and 67% Percoll, washed in media, resuspended in RPMI with 10% FBS, counted, and stained for flow cytometric analysis [38].

### Flow cytometric analysis

Lamina propria cells from SI of ΔdblGata or WT mice were stained with phycoerythrin (PE)- conjugated anti-c-Kit, fluorescein isothiocyanate (FITC)-conjugated anti-β7 integrin, Horizon V500-conjugated CD4, APC-Cy-7-conjugated anti-CD3e (BD Pharmingen, Mountain View, CA, USA), allophycocyanin (APC)-conjugated anti-IL-17RB, PE-Cy7-conjugated anti-FcεRIα (Biolegend, San Diego, CA, USA) and biotinylated anti-T1/ST2. Subsequently, cells were counterstained with PerCP-Cy5.5-conjugated monoclonal antibodies against lineage (Lin) markers (CD11b, CD11c, CD45R (BD Pharmingen, Mountain View, CA, USA) CD8α, Ly6G, and Pacific Blue labelled Streptavidin (Biolegend, San Diego, CA, USA)) before analysis with a FACS Canton II (BD Bioscience, Mountain View, CA, USA). CD4^+^ Th_2_ cells were identified as Lin^-^, CD3^+^, CD4^+^, and IL17RB^+^ populations. ILC2 cells were identified as Lin^-^, CD3^-^, CD4^-^, 1L17RB^+^, and c-Kit^-^ populations. MMC9 cells were identified as Lin^-^, CD3^-^, CD4^-^, 1L17RB^-^, c-Kit^+^, and FcεRIα^+^ populations as previously described [38]. All cytometric data was acquired using BD FACSCanto II and data analysis was performed using Flowjo software (FlowJo, Ashland, OR, USA).

### Bone marrow derived mast cell culture

Femur and tibia were harvested from age and gender matched WT and ΔdblGata mice and bone marrow cells were cultured in mIL-3 and mSCF (20ng/mL each) as previously described [39]. Mast cell maturation and purity was tracked weekly via FACS analysis for seven weeks. Cells were stained with phycoerythrin (PE)- conjugated anti-c-Kit (BD Pharmingen, Mountain View, CA, USA), PE-cy7-conjugated anti-FcεRIα (Biolegend, San Diego, CA, USA) and biotinylated anti-T1/ST2. Analysis was performed on FACS Canton II (BD Bioscience, Mountain View, CA, USA).

### Bone marrow mast cell degranulation assay

β-hexosaminidase assays were performed on the bone-marrow derived mast cells once they had reached maturation (≥85% c-Kit^+^ FcεRIα^+^ cells) as previously described [39]. Percent lysates were calculated by dividing treated WT and ΔdblGata BMMCs by their respective lysates and then multiplying by 100.

### Passive anaphylaxis

Mice were injected intravenously with 20μg/200μL of anti-IgE (IgG_2a_ mAb to mouse IgE; EM-95; obtained from Fred Finkelman, CCHMC) [40]. The severity of shock was assessed by means of rectal temperature change as previously described [32]. Blood was drawn intro heparinized capillary tube and centrifuged for 5 min at 9,500g. Hematocrit (percentage of packed red blood cell (RBC) volume) was calculated as the length of packed RBCs divided by the total length of serum and red cells in the capillary tube, multiplied by 100 [41, 42].

### Peritoneal wash

Naïve Balb/c and ΔdblGata mice were euthanized and their peritoneal cavity was flushed by injecting 10mL of PBS in 10% FBS into peritoneal cavity, massaging the abdomen, and then drawing out the fluid. Cells were kept on ice, centrifuged at 500g for 5 mins, and supernatant removed. Pellets were lysed with 5mL Red Blood Cell Lysis Buffer (Sigma-Aldrich, St. Louis, MO, USA) for 5 minutes at room temperature, 5mL of RPMI 1640 media added to neutralize lysis, and centrifuged for 5 mins. Pellet was re-suspended in media and counted. Cells were stained with phycoerythrin (PE)- conjugated anti-c-Kit (BD Pharmingen, Mountain View, CA, USA), PE-cy7-conjugated anti-FcεRIα (Biolegend, San Diego, CA, USA) and biotinylated anti-T1/ST2. They were subsequently counterstained with Pacific Blue labelled streptavidin. Analysis was performed on FACS Canton II (BD Bioscience, Mountain View, CA, USA) and analyzed using Flowjo (Flowjo Software, Ahsland, OR, USA).

### Statistical analysis

All data is represented with mean unless otherwise stated. In experiments comparing multiple experimental groups, statistical differences between groups were analyzed using the one/two-way ANOVA parametric test. In experiments comparing two experimental groups, statistical differences between groups were determined using a Student’s t-test or Wilcoxon-Mann-Whitney test. Results are considered significant at *P* ≤ 0.05. Spearman's rank coefficients were used to quantify the relations between percentage of CD4^+^ Th2 (IL17RB^+^) cells and level of mast cell activation (MCPT-1) following the eighth food challenge. All data was analyzed using Prism (GraphPad Software, San Diego, CA, USA).

## Results

### Reduced GATA-1 function alter *in vitro* bone marrow mast cell (BMMC) phenotype

**Fig 1:**
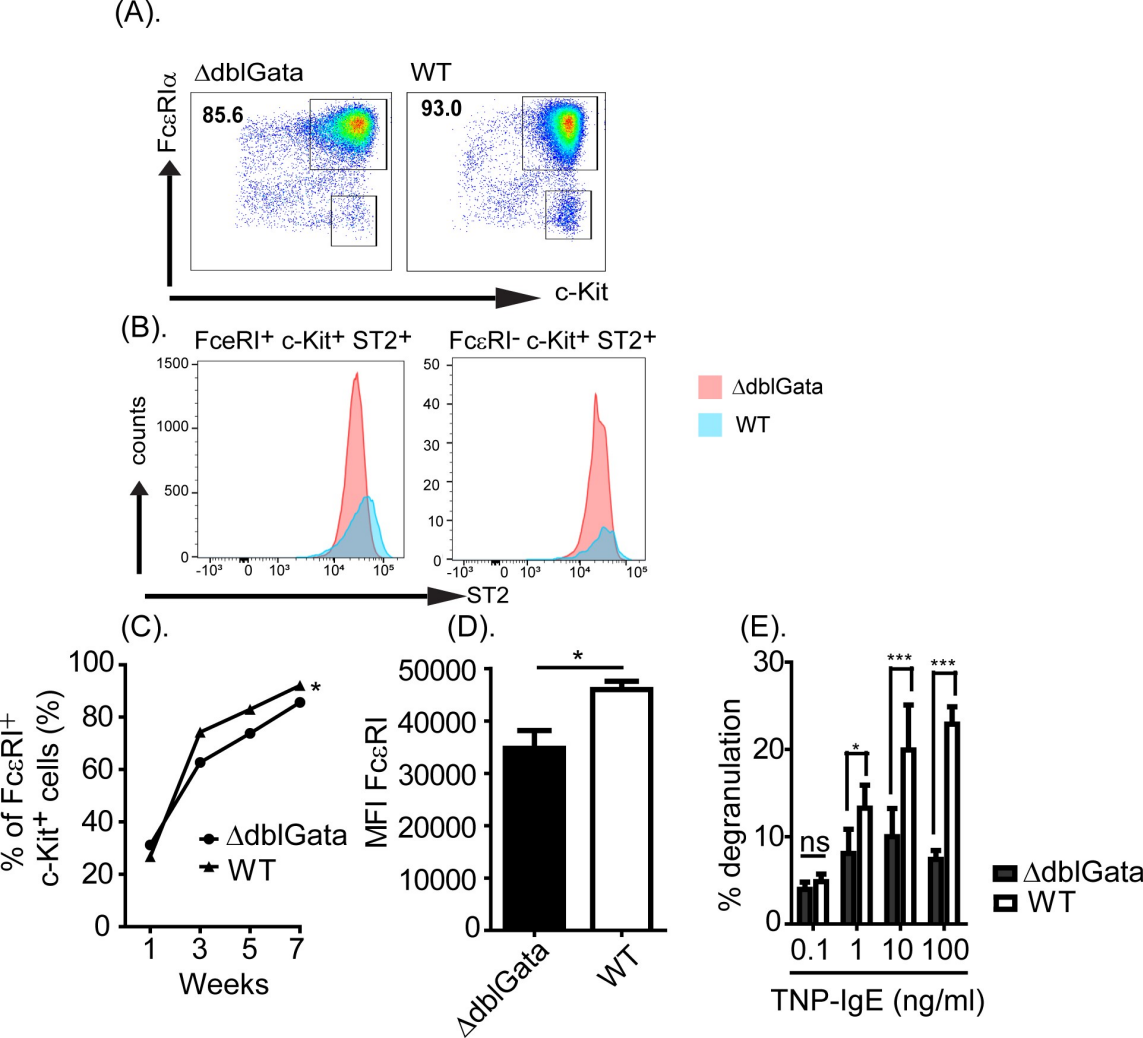
Bone marrow derived MC phenotype in ΔdblGata mice. (A) Representative flow cytometry plot of WT and ΔdblGata BMMCs (FcεRIα^+^ c-Kit^+^ cells) and (B) Histogram of ST2 expression of FcεRIα^+^ c-Kit^+^ and FcεRIα^-^ c-Kit^+^ cells from WT and ΔdblGata cultures at seven weeks of age. (C) The percentage of FcεRIα^+^ c-Kit^+^ cells in WT and ΔdblGata mice BM cultures over the seven week period. (D) The mean fluorescence intensity (MFI) of FcεRIα on c-Kit^+^ WT and ΔdblGata mature BMMCs. (E) The level of β-hexosaminidase activity following IgE-mediated degranulation of WT and ΔdblGata BMMCs. BM cells were isolated from 6–8 week old mice were cultured in the presence of IL-3 (20 ng/ml) and SCF (20 ng/ml) for seven weeks, and BMMCs were examined for FcεRIα, c-Kit, and ST2 expressions by flow cytometry analyses. 7 week-cultured WT and ΔdblGata BMMCs (5 × 10^6^/ml) were sensitized with IgE-TNP (0.1–100 ng/ml) and challenged with BSA-TNP and supernatant was assayed for β-hexosaminidase activity as described in materials and methods. Data represents mean ± SEM and is representative of three separate experiments. Statistical significance is * p ≤ .05, *** p < 0.001 compared with negative control (IgE and BSA-TNP negative). n.s. not significant.

Bone marrow cells from WT and ΔdblGata mice were cultured in mast cell growth conditions (IL-3 and SCF) for seven weeks and bone marrow-derived mast cell (BMMC) frequency and maturity were characterized by c-Kit, FcεRIα and ST2 analysis (Fig 1A and 1B). At seven weeks, the culture predominantly consisted of mature BMMCs (c-Kit^+^, FcεRIα^+^ and ST2^+^) (≥85%) with a low frequency of mast cell progenitors (c-Kit^+^, FcεRIα^-^ and ST2^+^) (Fig 1A and 1B). Notably, BMMC maturation was associated with high expression of FcεRIα and degranulation capacity (Fig 1D and 1E). In contrast, ΔdblGata cultures had a lower percentage of mature BMMCs (c-Kit^+^, FcεRIα^+^ and ST2^+^) by seven weeks and the BMMCs had reduced levels of FcεRIα expression and degranulation capacity (Fig 1A–1E). Based upon these studies, we concluded that GATA-1 signaling is dispensable for MC differentiation, but required for normal development *in vitro*.

### Role of GATA-1 signaling in passive IgE-mediated anaphylaxis

**Fig 2:**
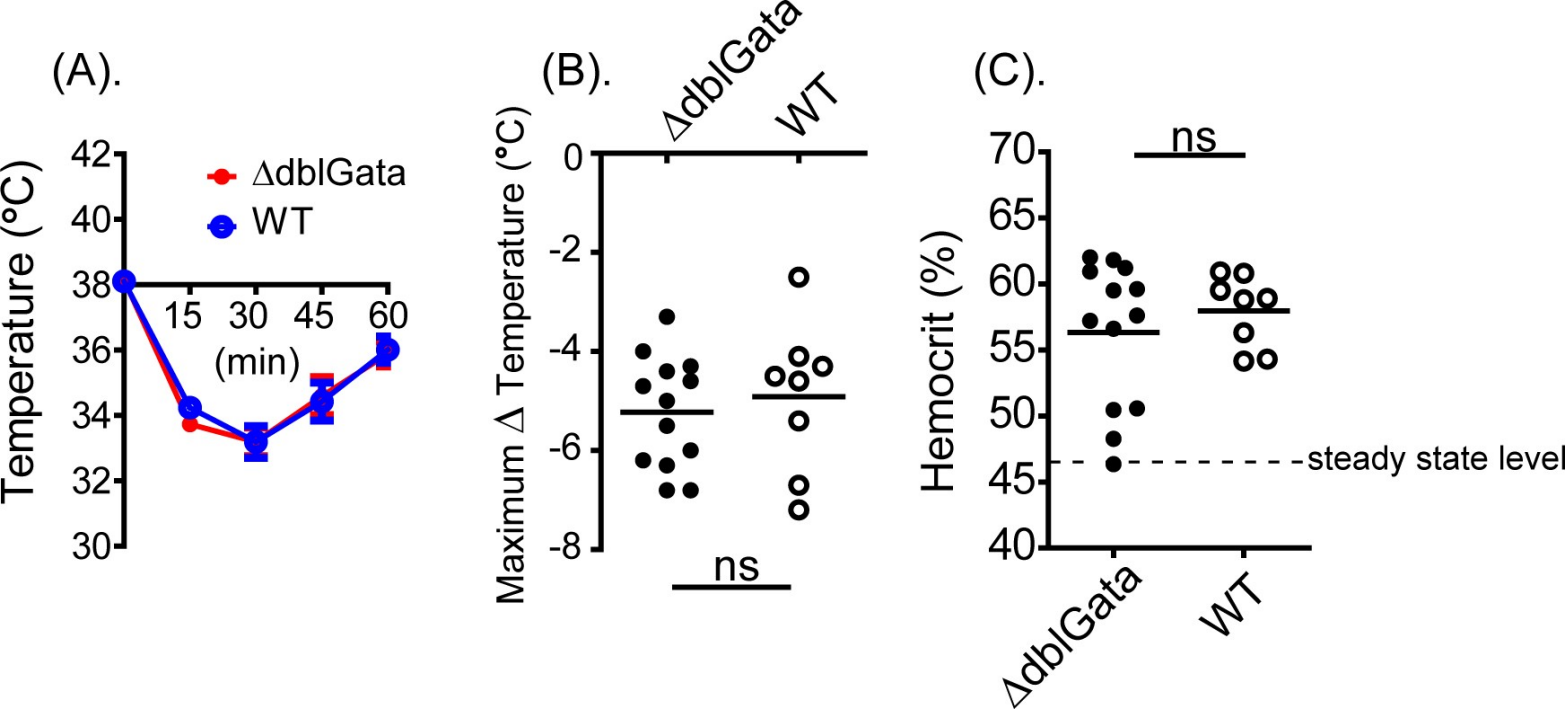
IgE-mediated passive anaphylaxis in WT and ΔdblGata mice. (A) Rectal temperature 0–60 minutes, (B) maximal temperature change at 30 minutes, and (C) hemocrit concentration at 60 minutes in anti-IgE-treated (20 μg/200 μl saline) WT and ΔdblGata mice. Data represent mean ± SEM. (A) represented as mean ± SEM; n = 3 or 4 mice per group per experiment pooled from three independent experiments. (B) and (C) line indicates mean and symbols represent individual mice. Dashed line indicates steady state hemaconcentration at baseline. n.s. not significant.

Given the reduced mast cell differentiation efficiency in ΔdblGata mice, we examined the requirement for GATA-1 signaling in IgE-mediated anaphylaxis. Administration of anti-IgE to WT mice induced hypovolemic shock as evidenced by ≥ 4°C loss in core body temperature and increased hemoconcentration (Fig 2A–2C). Surprisingly, in ΔdblGata mice, we observed a similar response to that of WT mice (Fig 2A–2C). Moreover, ΔdblGata mice experienced a comparable loss in body temperature and increased hemoconcentration following anti-IgE administration (Fig 2B and 2C). We concluded that reduced GATA-1 signaling does not impact passive IgE-mediated responses in mice at steady state.

### Requirement of GATA-1 signaling for steady state tissue MCs

**Fig 3:**
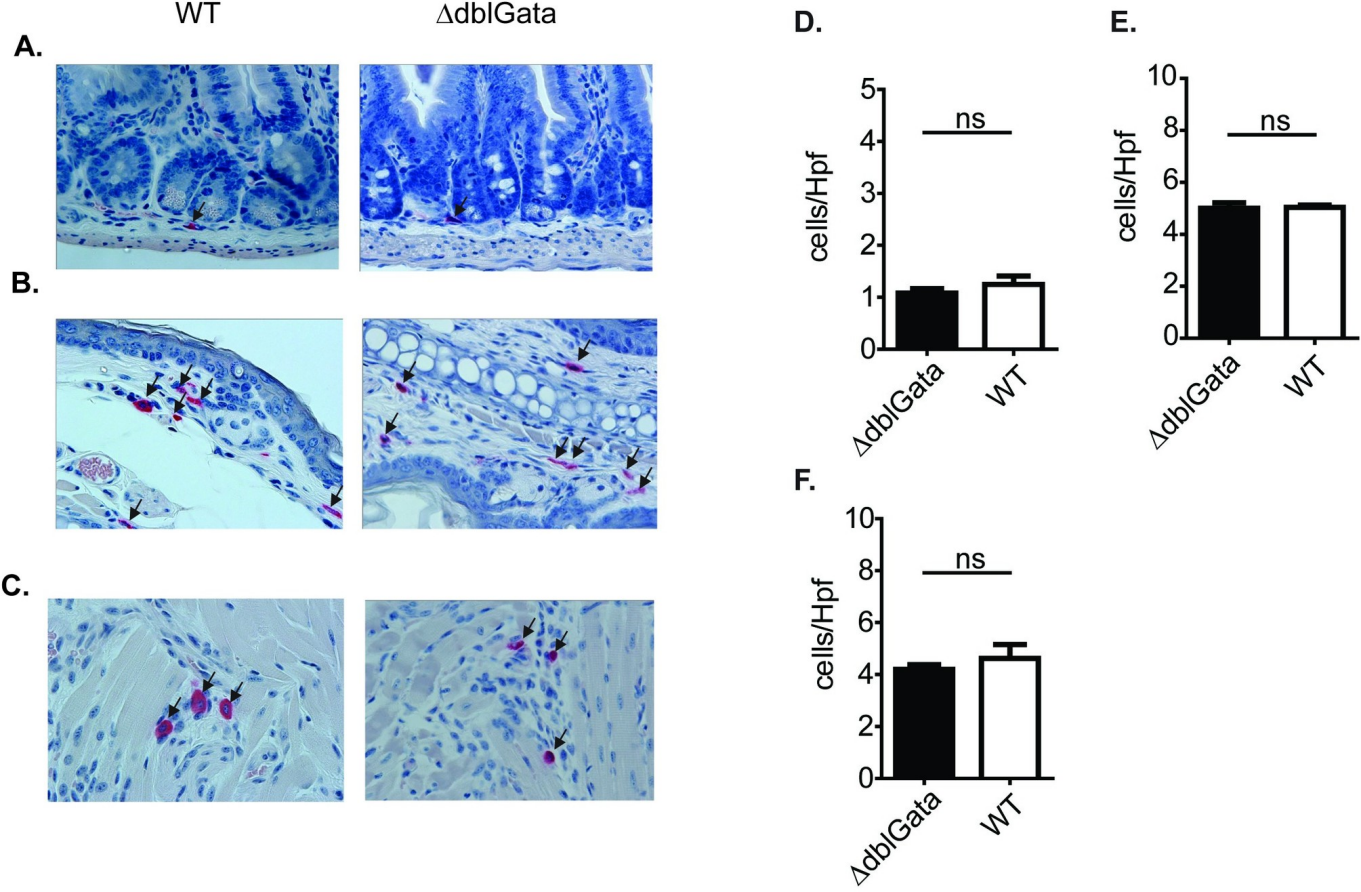
Systemic mast cells in WT and ΔdblGata mice at steady state. Representative photomicrographs and quantification of Mast cells/Hpf in: A and D) jejunum, (B and E) ear skin and (C and F) tongue of WT and ΔdblGata mice. Data represent mean ± SEM (n = 4–6) and is representative of two separate experiments. n.s. not significant.

Given the paradoxical observations of decreased FcεRI expression and degranulation capacity of ΔdblGata BMMCs, but comparable IgE-mediated responses *in vivo* compared to WT mice, we examined steady state tissue-resident mature mast cell levels in WT and ΔdblGata mice. Histological analyses of SI, ear, and tongue of WT and ΔdblGata mice revealed no significant differences in MC numbers (Fig 3A–3F). Analysis of peritoneal cavity revealed a 2-fold increase in peritoneal mast cell (pMC) levels in ΔdblGata mice compared to WT (S1 Fig). Notably, level of FcεRIα expression on pMCs was reduced in ΔdblGata mice when compared to mice (S1 Fig). Based upon these studies we concluded that a reduction of GATA-1 signaling does not impact SI, skin, and tongue MC levels, and increased pMC frequency.

### Requirement for GATA-1 signaling for IgE-MC-dependent responses *in vivo*

**Fig 4:**
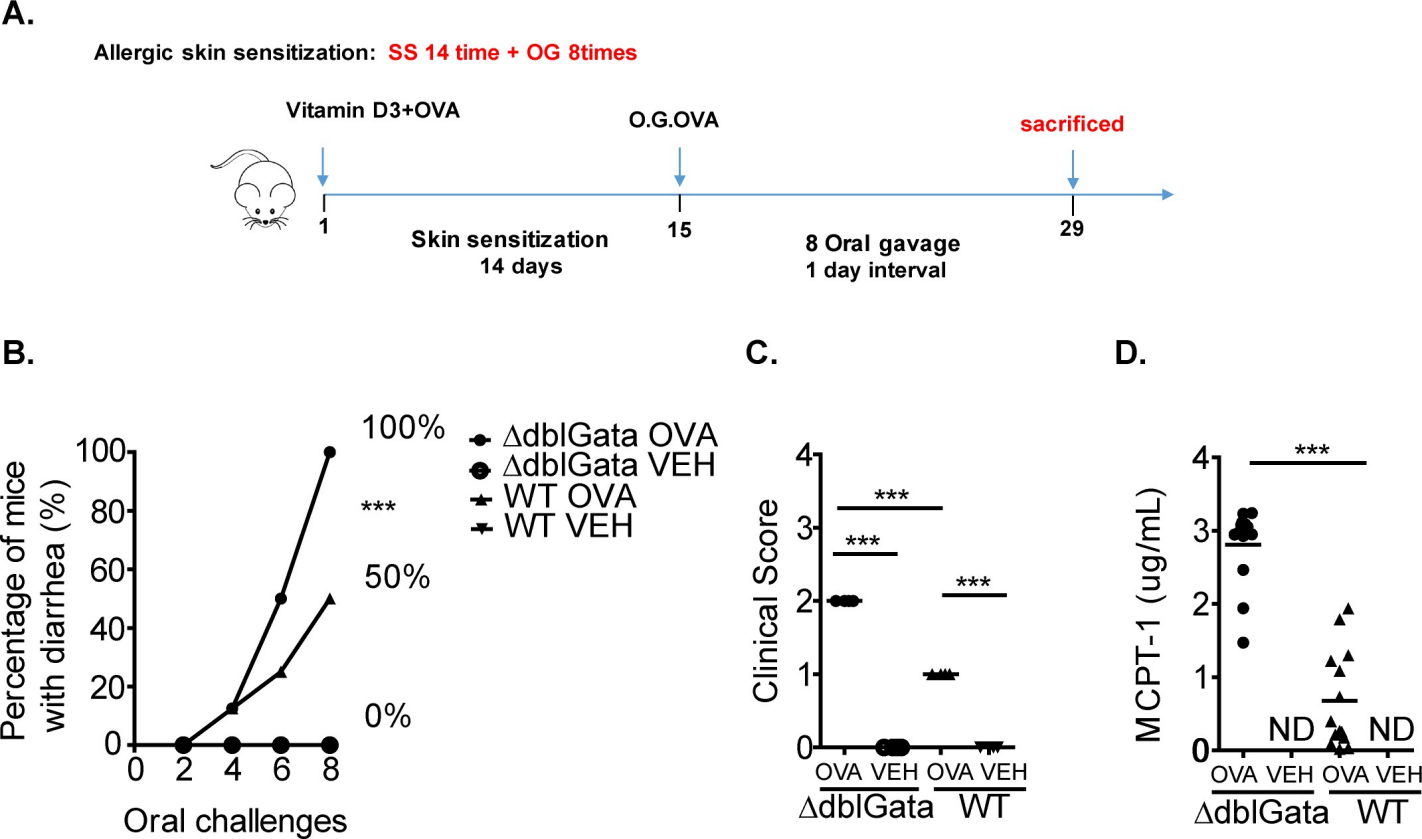
Active IgE-MC-dependent anaphylaxis in WT and ΔdblGata mice. (A) Oral antigen-triggered anaphylaxis experimental regime. (B) Diarrhea occurrence, (C) clinical score and (D) serum mast cell protease-1 (MCPT-1) levels in OVA-sensitized, i.g. Veh- or OVA-challenged BALB/c WT and ΔdblGata mice. The percentage in panel B represent the number of mice that experienced diarrhea over the number of total mice challenged as a percentage. Panels C and D were analyzed following eighth challenge of OVA-sensitized, i.g. Veh- or OVA-challenged mice. (C) and (D) line indicates mean and symbols represent individual mice. Values represent mean ± SEM; n = 6 to 10 mice per group. Statistical significance is *** p < 0.01. ND non detected.

To determine the contribution of GATA-1 signaling in MC-dependent inflammatory responses, we employed an active food allergy model [43]. Antigen sensitization and repetitive oral challenges of WT mice induced anaphylaxis as evidenced by diarrhea and clinical symptoms (Fig 4A–4C). Notably, development of symptoms of food-induced anaphylaxis was associated with MC activation (Fig 4D). To confirm that the food allergic response is IgE-dependent, WT mice that demonstrated evidence of food-induced anaphylaxis following the seventh oral gavage were administered either isotype control or rat anti-mouse IgE twenty-four hours prior to o.g. challenge eight. Administration of the activating rat IgG2a mAb to mouse IgE, EM-95, has been shown to induce a anaphylaxis characterized by decreased mobility and hypothermia [33] and subsequently desensitize mice to IgE-mediated responses for seventy two hours [34]. Food allergic WT mice receiving isotype control developed food allergy following challenge as evidenced by diarrhea. In contrast, mice that received EM-95 did not demonstrate evidence of allergic symptoms following oral gavage (% mice with evidence of Food Allergy following Challenge 7 / Challenge 8; isotype, 100% / 100%; EM-95, 100% / 16.6%, n = 3 and 6; p < 0.0001) confirming that the food allergic response is IgE-dependent. Sensitization and repeated antigen challenge of ΔdblGata mice also induced a food-induced anaphylactic response; surprisingly the ΔdblGata mice were more susceptible to IgE-MC-dependent food allergy. Moreover, only 50% of WT mice had diarrhea on the eighth challenge compared to 100% of ΔdblGata mice (Fig 4B). Notably, the ΔdblGata mice developed more severe symptoms following the eighth challenge including repetitive nose and ear scratching as well as lethargy and pilar erecti (Fig 4C). The heightened disease severity was associated with significantly increased SI mast cell numbers and mast cell activation (MCPT-1) as compared with WT mice (Fig 4D and S2 Fig). These data are consistent with previous reports demonstrating a positive correlation between SI mast cell levels and activation and severity of oral antigen-induced anaphylaxis [32]. We have previously reported that sensitization of mice to OVA via intraperitoneal injection and subsequent repetitive OVA oral gavage induces an IgE-MC-dependent anaphylactic response [32, 43]. To confirm the observed heightened responsiveness in ΔdblGata mice in an independent model, WT and ΔdblGata mice were sensitized to OVA and subsequently challenged by oral gavage. We show that consistent with the epicutaneous sensitization model, ΔdblGata mice demonstrated more severe food allergy phenotype with heightened intestinal mastocytosis (SI Mast cells / hpf; WT 149.6 ± 4.7 vs ΔdblGata 205.6 ± 7.5; n = 5 per group; mean ± SEM, p < 0.05) and mast cell activation (serum MCPT-1 levels (μg/ml): WT, 2.9 ± 1.4 vs ΔdblGata, 11.0 ± 0.9; n = 7 and 12 per group; mean ± SEM, p < 0.05). Collectively, employing two different models we show that ΔdblGata mice are more susceptible to IgE-MC-dependent responses in vivo.

### Enhanced IgE-MC mediated responses in ΔdblGata mice is associated with increased Type-2 response

**Fig 5:**
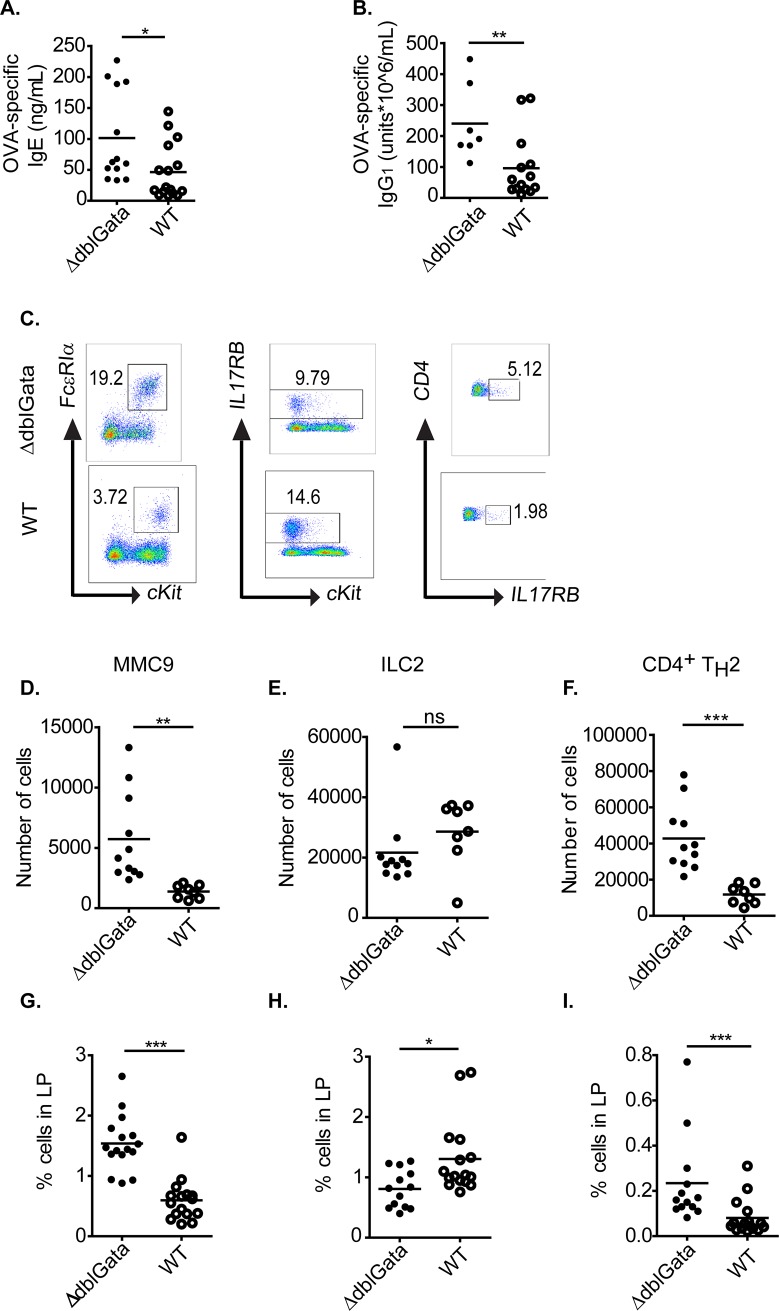
OVA-specific Ig, CD4^+^ T_H_2 cell, MMC9 and ILC2 frequency in SI of WT and ΔdblGata mice. (A and B) Antigen-specific IgE and IgG1 in serum, (C) detection and frequency of SI Lin^−^ST2^+^ FcεRIα^+^ c-Kit^+^ β7integrin^low^ MMC9 (D and G), Lin^-^IL-17RB^+^c-Kit^-^ ILC2s (E and H) and Lin^-^CD3^+^CD4^+^IL-17RB^+^ T_H_2 cells (F and I) from OVA-sensitized, i.g. OVA-challenged BALB/c WT and ΔdblGata mice. All analyses were performed following eighth challenge of OVA-sensitized, i.g. Veh- or OVA-challenged mice. A, B and D-I, line indicates mean and symbols represent individual mice. Statistical significance is * p ≤ .05, ** p ≤ .01, and *** p ≤ .001.

Type 2 immunity has been shown to be critical for the IgE-mast cell response in murine models of food allergy [3, 44, 45]. To determine whether increased food allergy susceptibility in ΔdblGata was associated with increase Type 2 immunity, we assessed antigen specific IgE and IgG1 levels (Fig 5A and 5B). Notably, while both WT and ΔdblGata mice generated antigen-specific IgE and IgG_1_ responses, ΔdblGata mice had significantly higher levels of both OVA-specific IgG_1_ and IgE compared to WT mice (Fig 5A). Given the relationship between antigen specific IgE and Type 2 immune response, we examined for presence of CD4^+^ T_H_2 cells (CD4^+^ IL17RB^+^) (Fig 5F and 5I) and ILC2 (Lin^-^, c-Kit^+^ IL17RB^+^) cells (Fig 5E and Fig 5H) within the SI of OVA-sensitized and challenged WT and ΔdblGata mice (Fig 5C). We identified a significant increase of CD4^+^ T_H_2 cells in the SI of ΔdblGata mice as compared to WT mice (Fig 5F and 5I). Although there was a significant decrease in the population percentage of ILC2 cells, the difference was not significant in total cell numbers between WT and ΔdblGata mice (Fig 5E and 5H). MMC9 cells have been recently shown to be the main source of IL-9 in the SI [38] which promotes mast cell maturation and intestinal mastocytosis and drives IgE-MC-dependent response [38, 43]. Analysis of MMC9 levels in WT and ΔdblGata mice revealed that OVA sensitization and repeated challenge induced SI MMC9 levels in both WT and ΔdblGata mice (Fig 5D and 5G). Consistent with elevated mature MC levels in ΔdblGata mice (S2 Fig), levels of SI MMC9 cells were also increased in ΔdblGata mice compared with OVA-challenged WT mice (Fig 5D and 5G). To determine whether the observed differences in Type-2 immunity and SI MMC9 phenotype in ΔdblGata mice was due to homeostatic variances, we examined SI immune profile of WT and ΔdblGata mice at steady state. Notably, levels of total serum IgE and CD4^+^ Th_2_, ILC2, and MMC9 cells levels in the SI were comparable between WT and ΔdblGata mice at steady state (S3 Fig). These studies suggest that ΔdblGata mice have normal Type-2 immune cells in the SI at steady state and that antigen challenge of these mice leads to heightened SI CD4^+^ Type-2 immune responses.

## Discussion

In the present study, we have investigated the requirement of GATA-1 signaling in the development and maturation of MCs and IgE-induced MC function *in vitro* and *in vivo*. We show that diminished GATA-1 function reduced BMMC maturation rate, level of FcεRI expression and degranulation capacity. However, *in vivo* analyses revealed that diminutive GATA-1 activity does not impact tissue MC frequency nor MC functionality at steady state. Surprisingly, employing models of passive and active IgE-mediated anaphylaxis, we show that diminished GATA-1 functionality has no impact on IgE-MC-dependent responses in the naïve state, however, antigen sensitization models revealed that low GATA-1 activity amplifies IgE-MC-dependent responses and that the increased response appeared to be associated with a stronger pro-Type 2 response associated with increased antigen-specific IgE and CD4^+^ Th_2_ response. Despite, GATA-1 intrinsic requirement for MC maturation and IgE-functionality *in vitro*, *in vivo* analyses reveals heightened MC functionality and IgE-MC-dependent immune responses.

There has been a number of studies employing various transgenic and gene-deficient mice defining the involvement of GATA-1 in MC biology [26–28, 46] and these studies have identified a complex role for GATA-1 in the differentiation, maturation and functionality of MCs. Some studies have reported that GATA-1 is not required for MC differentiation and maturation, however other studies report an important role for MC maturation and functionality [26, 27, 46]. There has been a number of potential explanations for these discrepancies related to the level of GATA-1 promoter inactivation in the various genetically modified mice. Moreover, MC-intrinsic differences in GATA-1 activity (GATA^low^ versus ΔdblGata mice) contributes to the differences in surface expression of FcεRI on BMMCs [26, 28]. These studies are supported by the observations of GATA-1 binding to the promoter region of *FcεRI*, *ckit* and *cpa3* genes in BMMC’s [47, 48] and the identification of GATA-1 binding motifs in the 5’-end of group A tryptase [27]. Collectively these studies indicate that GATA-1 is dispensable for MC differentiation [27] however, GATA-1 plays a conserved role in later stage MC maturation through its involvement in transcriptional regulation of expression of several MC specific genes including *FcεRIα*, *FcεRIβ* and *Cpa3* [49–51]. MC differentiation and maturation involves a complex interplay by several transcription factors including PU.1, MTIF, STAT5, C/EPBα, and GATA-2 [15–19]. Unlike following GATA-1 deletion, GATA-2 deletion leads to loss of MC lineage specification [52] suggesting that GATA-2 plays a more dominant role in mast cell differentiation. Elegant studies by Ohneda and colleagues revealed that GATA-2 is required for cell lineage specification and the maintenance of mast cells in the differentiated state, whereas, GATA-1 regulates expression of MC specific group A tryptase gene expression and mast cell maturation [27].

The observed *in vitro* effects on diminished GATA-1 signaling on BMMC FcεRI expression and degranulation capacity prompted us to examine the impact of diminished GATA-1 signaling on IgE-MC dependent responses. To our surprise we observed no difference in IgE-mediated MC-dependent shock responses between WT and ΔdblGata mice. We speculated that the observed discrepancy between the *in vitro* and *in vivo* observations could be explained by heightened MC activation following IgE crosslinking or altered steady state tissue MC levels. However, examination of the surrogate MC activation marker (MCPT-1) revealed comparable level of MC activation between groups and peripheral tissue MC levels at steady state in ΔdblGata mice were not significantly different to WT mice. The observed similarities in distribution or frequency of MC populations in peripheral tissues is consistent with previous studies employing ΔdblGata and Gata1^-/γ^ mice [27]. To our knowledge this is the first *in vivo* examination of MC function *in vivo* in GATA^low^ mice. Interestingly, previous reports suggest that IgE-FcεRI-MC-dependent anaphylaxis in mice is dependent on connective tissue mast cells (CTMC) and histamine [53]. Examination of frequency and phenotype of MCs in the peritoneal lavage of ΔdblGata mice revealed higher MC numbers and reduced expression of FcεRI. The increased level of MCs in peritoneal lavage is consistent with the observed higher incidence of immature MCs (c-kit^high^ FcεRI^low^) observed in peritoneal lavage of GATA-1^low^ mice [54]. Previous studies have reported a central role for GATA-2 and MITF in CTMC differentiation and IgE-MC-mediated anaphylaxis [55]. MITF and GATA-2 critically regulate histidine decarboxylase (HDC) gene expression and histamine synthesis [55] whereas GATA-1’s effects on MC is likely restricted to regulation of expression of specific MC genes related to group A Tryptase gene expression (e.g. Mcpt-6) which are not necessary for IgE-FcεRI-MC-dependent anaphylaxis.

Employing our active models of IgE-MC-dependent anaphylaxis [32], we revealed heightened IgE-MC-dependent shock response during diminished GATA-1 activity. This is in contrast to a recent study that demonstrated no difference in IgE-mediated anaphylactic responses between ΔdblGata and WT mice employing a similar sensitization and challenge model [56]. We speculate that the differences observed between the two studies relates to the number of oral gavage challenges performed. Hussian and colleagues performed two oral gavage challenges whereas we performed eight repetitive challenges. The increased number of challenges elicits a stronger SI Th2 response and mastocytosis and following oral gavage challenge a stronger IgE-MC-response with increased severity [3, 32, 38, 57]. Albeit not significantly different, Hussain and colleagues did observe heightened level of SI MCs in ΔdblGata mice as compared to WT mice following two challenges [56].

We currently do not fully understand how diminutive levels of GATA-1 could lead to heightened *in vivo* IgE-MC-dependent reactions. We do show that the limited GATA-1 functionality does not impact steady state tissue MC levels or SI CD4^+^ T cell, ILC2 and MMC9 numbers suggesting that the observed phenotype is not attributed to homeostatic defects. Antigen sensitization and challenge of ΔdblGata mice did lead to increased antigen-induced CD4^+^ Th2 cells and antigen-specific IgE. CD4^+^ Th2 responses are required for the development of IgE-MC-dependent food-induced anaphylaxis and CD4^+^ Th2 cell levels do positively correlate with food allergen-induced MC activation (MCPT-1) (r = 0.57, p < 0.05). However, GATA-1 activity is conserved in CD4^+^ Th2, ILC2 and iMMC9 cells and altered GATA-1 functionality is known to modulate cellular functionality and thus anyone of these populations could contribute to the described allergic outcomes.

The ΔdblGata mice are eosinophil deficient and also possess a defect in basophil frequency and function [58]. Previous studies have reported a role for basophil-derived IL-4 in epicutaneous food antigen sensitization and food allergy in mice [59]. Furthermore, eosinophils have been observed to infiltrate the large intestine in mice with allergy induced diarrhea [60] and interactions between eosinophils and mast cells lead to mast cell degranulation [61–63]. Our demonstration of antigen sensitization and the development of IgE-dependent reaction in ΔdblGata mice comparable to WT mice suggests that eosinophils and basophils are not necessary for intraperitoneal antigen sensitization and development of IgE-MC-mediated food allergy and anaphylaxis.

In conclusion, mice with diminished GATA-1 function reveal a complex role for GATA-1 in MC maturation and functionality. In vitro, reduced GATA-1 activity leads to impairment of MC maturation and IgE-mediated degranulation supporting an important role for GATA-1 in MC function. However, in vivo analyses reveal that the MC-intrinsic GATA-1 requirement in maturation and degranulation does not necessarily impact IgE-MC-dependent immune responses. These studies reveal the complexity of immune signaling pathways with the existence of compensatory processes that can independently alter cell intrinsic requirements via cell extrinsic processes.

## Supporting information

S1 Fig Peritoneal wash mast cells. Peritoneal FcεRIα^+^ c-Kit^+^ mast cell frequency and (B) mean fluorescence intensity (MFI) of FcεRIα on Peritoneal c-Kit^+^ mast cells. (A) and (B) line indicates mean and symbols represent individual mice and is representative of three separate experiments. Statistical significance is ** p ≤ .01.
(AI) Click here for additional data file.

S2 Fig Increased intestinal mastocytosis in allergy induced ΔdblGata mice. (A–C) Representative photomicrographs and quantification of Mast cells/mm^2^. (C) line indicates mean and symbols represent individual mice. Data representative of four independent experiments. Magnification x50 (A and B, left panels) and x200 (A and B, right panels). Statistical significance is *** p ≤ .001.
(AI) Click here for additional data file.

S3 Fig Total IgE, CD4+ TH2 cell, MMC9 and ILC2 frequency in SI of WT and ΔdblGata mice at steady state. (A) Total IgE in serum. (B) detection and frequency of SI Lin^−^ST2^+^ FcεRIα^+^ c-Kit^+^ β7integrin^low^ MMC9, (C) Lin^-^IL-17RB^+^c-Kit^-^ ILC2s, and (D) Lin^-^CD3^+^CD4^+^IL-17RB^+^ T_H_2 cells from BALB/c WT and ΔdblGata mice at steady state. (A-D) line indicates mean and symbols represent individual mice. Data representative of three separate experiments. n.s. not significant.
(AI) Click here for additional data file.

## Abbreviations

MC: Mast Cell
MMC9: Interleukin-9 secreting mucosal mast cells
MCPt-1: Mouse mast cells protease-1
ILC2: Type 2 Innate Lymphoid Cells
OVA: ovalbumin
HBSS: Hanks balanced salt solution
SCF: stem cell factor
WT: Wild type
SI: Small intestine
BMMC: Bone marrow mast cell culture
pMC: peritoneal Mast Cell
CTMC: Connective tissue mast cells

## References

[1] Galli SJ, Tsai M. IgE and mast cells in allergic disease. Nature medicine. 2012;18(5):693–704. Epub 2012/05/09. 10.1038/nm.2755 . 22561833 PMC3597223

[2] Bartnikas LM, Gurish MF, Burton OT, Leisten S, Janssen E, Oettgen HC, et al Epicutaneous sensitization results in IgE-dependent intestinal mast cell expansion and food-induced anaphylaxis. J Allergy Clin Immunol. 2013;131(2):451–60 e1-6. Epub 2013/02/05. 10.1016/j.jaci.2012.11.032 23374269 PMC3587010

[3] Brandt EB, Munitz A, Orekov T, Mingler MK, McBride M, Finkelman FD, et al Targeting IL-4/IL-13 signaling to alleviate oral allergen-induced diarrhea. J Allergy Clin Immunol. 2009;123(1):53–8. 10.1016/j.jaci.2008.10.001 . 18996576 PMC4121593

[4] Benede S, Berin MC. Mast cell heterogeneity underlies different manifestations of food allergy in mice. PLoS One. 2018;13(1):e0190453 Epub 2018/01/26. 10.1371/journal.pone.0190453 29370173 PMC5784907

[5] MacGlashan D Jr. IgE receptor and signal transduction in mast cells and basophils. Current opinion in immunology. 2008;20(6):717–23. 10.1016/j.coi.2008.08.004 . 18822373

[6] Berin MC. Pathogenesis of IgE‐mediated food allergy. Clinical & Experimental Allergy. 2015;45(10):1483–96. 10.1111/cea.12598 26215729

[7] Hogan SP, Wang YH, Strait R, Finkelman FD. Food-induced Anaphylaxis: Mast Cells as Modulators of Anaphylactic Severity. Seminars in immunopathology. 2012;34(5):643–53. 10.1007/s00281-012-0320-1 PMC3924961. 22926692 PMC3924961

[8] Gupta RS, Springston EE, Warrier MR, Smith B, Kumar R, Pongracic J, et al The prevalence, severity, and distribution of childhood food allergy in the United States. Pediatrics. 2011;128(1):e9–17. Epub 2011/06/22. 10.1542/peds.2011-0204 . 21690110

[9] Gupta R, Holdford D, Bilaver L, Dyer A, Holl JL, Meltzer D. The economic impact of childhood food allergy in the United States. JAMA pediatrics. 2013;167(11):1026–31. 10.1001/jamapediatrics.2013.2376 . 24042236

[10] Schwartz LB, Yunginger JW, Miller J, Bokhari R, Dull D. Time course of appearance and disappearance of human mast cell tryptase in the circulation after anaphylaxis. J Clin Invest. 1989;83(5):1551–5. 10.1172/JCI114051 . 2468689 PMC303860

[11] Brown SG, Stone SF, Fatovich DM, Burrows SA, Holdgate A, Celenza A, et al Anaphylaxis: clinical patterns, mediator release, and severity. J Allergy Clin Immunol. 2013;132(5):1141–9 e5. Epub 2013/08/07. 10.1016/j.jaci.2013.06.015 . 23915715

[12] Vadas P, Gold M, Perelman B, Liss GM, Lack G, Blyth T, et al Platelet-Activating Factor, PAF Acetylhydrolase, and Severe Anaphylaxis. New England Journal of Medicine. 2008;358(1):28–35. 10.1056/NEJMoa070030 . 18172172

[13] Finkelman FD. Anaphylaxis: lessons from mouse models. The Journal of allergy and clinical immunology. 2007;120(3):506–15; quiz 16–7. 10.1016/j.jaci.2007.07.033 . 17765751

[14] Dombrowicz D, Flamand V, Brigman KK, Koller BH, Kinet J. Abolition of anaphylaxis by targetted disruption of the high affinity immunoglobulin E receptor a chain gene Cell. 1993;75:969–76. Epub December 3 1993. 10.1016/0092-8674(93)90540-7 8252632

[15] Baba Y, Maeda K, Yashiro T, Inage E, Niyonsaba F, Hara M, et al Involvement of PU.1 in mast cell/basophil-specific function of the human IL1RL1/ST2 promoter. Allergology international: official journal of the Japanese Society of Allergology. 2012;61(3):461–7. 10.2332/allergolint.12-OA-0424 . 22824976

[16] Barnstein BO, Li G, Wang Z, Kennedy S, Chalfant C, Nakajima H, et al Stat5 Expression Is Required for IgE-Mediated Mast Cell Function. The Journal of Immunology. 2006;177(5):3421–6. 10.4049/jimmunol.177.5.3421 16920984

[17] Ohmori S, Moriguchi T, Noguchi Y, Ikeda M, Kobayashi K, Tomaru N, et al GATA2 is critical for the maintenacnce of cellular identity in differentiated mast cells derived from mouse bone marrow. Blood. 2015;125:3306–15. 10.1182/blood-2014-11-612465 25855601

[18] Arinobu Y, Iwasaki H, Gurish MF, Mizuno S, Shigematsu H, Ozawa H, et al Developmental checkpoints of the basophil/mast cell lineages in adult murine hematopoiesis. Proceedings of the National Academy of Sciences of the United States of America. 2005;102(50):18105–10. 10.1073/pnas.0509148102 16330751 PMC1312421

[19] Morii E, Oboki K, Ishihara K, Jippo T, Hirano T, Kitamura Y. Roles of MITF for development of mast cells in mice: effects on both precursors and tissue environments. Blood. 2004;104(6):1656 10.1182/blood-2004-01-0247 15172970

[20] Kulessa H, Frampton J, Graf T. GATA-1 reprograms avian myelomonocytic cell lines into eosinophils, thromboblasts, and erythroblasts. Genes & Development. 1995;9(10):1250–62. 10.1101/gad.9.10.1250 7758949

[21] Martin DIK, Zon LI, Mutter G, Orkin SH. Expression of an erythroid transcription factor in megakaryocytic and mast cell lineages. Nature. 1990;344:444 10.1038/344444a0 2320112

[22] Wakabayashi J, Yomogida K, Nakajima O, Yoh K, Takahashi S, Engel JD, et al GATA‐1 testis activation region is essential for Sertoli cell‐specific expression of GATA‐1 gene in transgenic mouse. Genes to Cells. 2003;8(7):619–30. 10.1046/j.1365-2443.2003.00658.x 12839622

[23] Fujiwara Y, Browne C, Cunniff K, Goff SC, Orkin SH. Arrested development of embryonic red cell precursors in mouse embryos lacking transcription factor GATA-1. Proc Natl Acad Sci USA. 1996;93:12355–8. 10.1073/pnas.93.22.12355 8901585 PMC37995

[24] Takahashi S, Onodera K, Motohashi H, Suwabe N, Hayashi N, Yanai N, et al Arrest in primitive erythroid cell development caused by promoter-specific disruption of the GATA1 gene. The Journal of Biological Chemistry. 1997;272:12611–5 10.1074/jbc.272.19.12611 9139715

[25] Shivdasani RA, Fujiwara Y, McDevitt MA, Orkin SH. A lineage-selective knockout establishes the critical role of transcription factor GATA-1 in megakaryocyte growth and platelet development. The EMBO Journal. 1997;16(13):3965–73. 10.1093/emboj/16.13.3965 9233806 PMC1170020

[26] Migliaccio AR, Rana RA, Sanchez M, Lorenzini R, Centurione L, Bianchi L, et al GATA-1 as a Regulator of Mast Cell Differentiation Revealed by the Phenotype of the GATA-1lowMouse Mutant. The Journal of Experimental Medicine. 2003;197(3):281–96. 10.1084/jem.20021149 12566412 PMC2193836

[27] Ohneda K, Moriguchi T, Ohmori Sy, Ishijima Y, Satoh H, Philipsen S, et al Transcription Factor GATA1 Is Dispensable for Mast Cell Differentiation in Adult Mice. Molecular and cellular biology. 2014;34(10):1812–26. 10.1128/MCB.01524-13 PMC4019035. 24615013 PMC4019035

[28] Yu C, Cantor AB, Yang H, Browne C, Wells RA, Fujiwara Y, et al Targeted Deletion of a High-Affinity GATA-binding Site in the GATA-1 Promoter Leads to Selective Loss of the Eosinophil Lineage In Vivo. The Journal of Experimental Medicine. 2002;195(11):1387–95. 10.1084/jem.20020656 12045237 PMC2193547

[29] Humbles AA, Lloyd CM, McMillan SJ, Friend DS, Xanthou G, McKenna EE, et al A critical role for eosinophils in allergic airway remodeling. Science. 2004;305:1776–9. 10.1126/science.1100283 15375268

[30] Tsai SF, Strauss E, Orkin SH. Functional analysis and in vivo footprinting implicate the erythroid transcription factor GATA-1 as a positive regulator of its own promoter. Genes & Development. 1991;5(6):919–31. 10.1101/gad.5.6.919 2044960

[31] Trainor CD, Omichinski JG, Vandergon TL, Gronenborn AM, Clore GM, Felsenfeld G. A palindromic regulatory site within vertebrate GATA-1 promoters requires both zinc fingers of the GATA-1 DNA-binding domain for high-affinity interaction. Molecular and cellular biology. 1996;16(5):2238–47. PMC231211. 10.1128/mcb.16.5.2238 8628290 PMC231211

[32] Ahrens R, Osterfeld H, Wu D, Chen CY, Arumugam M, Groschwitz K, et al Intestinal mast cell levels control severity of oral antigen-induced anaphylaxis in mice. The American journal of pathology. 2012;180(4):1535–46. Epub 2012/02/11. 10.1016/j.ajpath.2011.12.036 22322300 PMC3354589

[33] Strait RT, Morris SC, Smiley K, Urban JF Jr., Finkelman FD. IL-4 exacerbates anaphylaxis. J Immunol. 2003;170(7):3835–42. 10.4049/jimmunol.170.7.3835 12646651

[34] Khodoun MV, Kucuk ZY, Strait RT, Krishnamurthy D, Janek K, Lewkowich I, et al Rapid polyclonal desensitization with antibodies to IgE and FcepsilonRIalpha. J Allergy Clin Immunol. 2013;131(6):1555–64. Epub 2013/05/02. 10.1016/j.jaci.2013.02.043 . 23632296 PMC4341981

[35] Li X-m, Schofield BH, Huang C-K, Kleiner GI, Sampson HA. A murine model of IgE-mediated cow's milk hypersensitivity. Journal of Allergy and Clinical Immunology. 1999;103(2):206–14. 10.1016/S0091-6749(99)70492-6 9949309

[36] Brandt EB, Strait RT, Hershko D, Wang Q, Muntel EE, Scribner TA, et al Mast cells are required for experimental oral allergen–induced diarrhea. Journal of Clinical Investigation. 2003;112(11):1666–77. 10.1172/JCI19785 14660743 PMC281649

[37] Forbes E, Smart VE, D’Aprile A, Henry P, Yang M, Matthaei KI, et al T helper-2 immunity regulates bronchial hyperresponsiveness in eosinophil-associated gastrointestinal disease in mice. Gastroenterology. 2004;127(1):105–18. 10.1053/j.gastro.2004.03.057 15236177

[38] Chen CY, Lee JB, Liu B, Ohta S, Wang PY, Kartashov AV, et al Induction of Interleukin-9-Producing Mucosal Mast Cells Promotes Susceptibility to IgE-Mediated Experimental Food Allergy. Immunity. 2015;43(4):788–802. 10.1016/j.immuni.2015.08.020 26410628 PMC4618257

[39] Kuehn HS, Radinger M, Gilfillan AM. Current Protocols in Immunology. 2010:7 38.1–7..9. 10.1002/0471142735.im0738s91 21053305 PMC2982193

[40] Yamani A, Wu D, Waggoner L, Noah T, Koleske AJ, Finkelman F, et al The vascular endothelial specific IL-4 receptor alpha–ABL1 kinase signaling axis regulates the severity of IgE-mediated anaphylactic reactions. Journal of Allergy and Clinical Immunology. 2017 10.1016/j.jaci.2017.08.046 PMC5957775 29157947

[41] Strait RT, Morris SC, Smiley K, Urban JF, Finkelman FD. IL-4 Exacerbates Anaphylaxis. The Journal of Immunology. 2003;170(7):3835 10.4049/jimmunol.170.7.3835 12646651

[42] Strait RT, Morris SC, Finkelman FD. IgG-blocking antibodies inhibit IgE-mediated anaphylaxis in vivo through both antigen interception and FcγRIIb cross-linking. The Journal of clinical investigation. 2006;116(3):833–41. 10.1172/JCI25575 16498503 PMC1378186

[43] Osterfeld H, Ahrens R, Strait R, Finkelman FD, Renauld JC, Hogan SP. Differential roles for the IL-9/IL-9 receptor alpha-chain pathway in systemic and oral antigen-induced anaphylaxis. J Allergy Clin Immunol. 2010;125(2):469–76 e2. Epub 2010/02/18. 10.1016/j.jaci.2009.09.054 . 20159257 PMC4259249

[44] Koyasu S, Moro K. Type 2 innate immune responses and the natural helper cell. Immunology. 2011;132(4):475–81. 10.1111/j.1365-2567.2011.03413.x PMC3075501. 21323663 PMC3075501

[45] Deo SS, Mistry KJ, Kakade AM, Niphadkar PV. Role played by Th2 type cytokines in IgE mediated allergy and asthma. Lung India: Official Organ of Indian Chest Society. 2010;27(2):66–71. 10.4103/0970-2113.63609 PMC2893428. 20616938 PMC2893428

[46] Nishiyama C, Ito T, Nishiyama M, Masaki S, Maeda K, Nakano N, et al GATA-1 is required for expression of Fc{epsilon}RI on mast cells: analysis of mast cells derived from GATA-1 knockdown mouse bone marrow. International immunology. 2005;17(7):847–56. 10.1093/intimm/dxh278 . 15967781

[47] Tripic T, Deng W, Cheng Y, Zhang Y, Vakoc CR, Gregory GD, et al SCL and associated proteins distinguish active from repressive GATA transcription factor complexes. Blood. 2009;113(10):2191–201. 10.1182/blood-2008-07-169417 PMC2652367. 19011221 PMC2652367

[48] Inage E, Kasakura K, Yashiro T, Suzuki R, Baba Y, Nakano N, et al Critical Roles for PU.1, GATA1, and GATA2 in the expression of human FcepsilonRI on mast cells: PU.1 and GATA1 transactivate FCER1A, and GATA2 transactivates FCER1A and MS4A2. Journal of immunology. 2014;192(8):3936–46. 10.4049/jimmunol.1302366 . 24639354

[49] Nishiyama C, Yokota T, Okumura K, Ra C. The Transcription Factors Elf-1 and GATA-1 Bind to Cell-Specific Enhancer Elements of Human High-Affinity IgE Receptor α-Chain Gene. The Journal of Immunology. 1999;163(2):623 10395650

[50] Maeda K, Nishiyama C, Tokura T, Akizawa Y, Nishiyama M, Ogawa H, et al Regulation of Cell Type-Specific Mouse FcεRI β-Chain Gene Expression by GATA-1 Via Four GATA Motifs in the Promoter. The Journal of Immunology. 2003;170(1):334 10.4049/jimmunol.170.1.334 12496417

[51] Zon LI, Gurish MF, Stevens RL, Mather C, Reynolds DS, Austen KF, et al GATA-binding transcription factors in mast cells regulate the promoter of the mast cell carboxypeptidase A gene. Journal of Biological Chemistry. 1991;266(34):22948–53. 1744088

[52] Tsai F-Y, Orkin SH. Transcription Factor GATA-2 Is Required for Proliferation/Survival of Early Hematopoietic Cells and Mast Cell Formation, But Not for Erythroid and Myeloid Terminal Differentiation. Blood. 1997;89(10):3636 9160668

[53] Li Y, Liu B, Harmacek L, Long Z, Liang J, Lukin K, et al The transcription factors GATA2 and microphthalmia-associated transcription factor regulate Hdc gene expression in mast cells and are required for IgE/mast cell-mediated anaphylaxis. J Allergy Clin Immunol. 2017;24(17):32917–2. 10.1016/j.jaci.2017.10.043 PMC6291018 29277702

[54] Migliaccio AR, Rana RA, Sanchez M, Lorenzini R, Centurione L, Bianchi L, et al GATA-1 as a regulator of mast cell differentiation revealed by the phenotype of the GATA-1low mouse mutant. J Exp Med. 2003;197(3):281–96. 10.1084/jem.20021149 12566412 PMC2193836

[55] Li Y, Liu B, Harmacek L, Long Z, Liang J, Lukin K, et al The transcription factors GATA2 and microphthalmia-associated transcription factor regulate Hdc gene expression in mast cells and are required for IgE/mast cell–mediated anaphylaxis. Journal of Allergy and Clinical Immunology. 2017 10.1016/j.jaci.2017.10.043 29277702 PMC6291018

[56] Hussain M, Borcard L, Walsh KP, Pena Rodriguez M, Mueller C, Kim BS, et al Basophil-derived IL-4 promotes epicutaneous antigen sensitization concomitant with the development of food allergy. Journal of Allergy and Clinical Immunology. 2018;141(1):223–34.e5. 10.1016/j.jaci.2017.02.035 28390860

[57] Forbes EE, Groschwitz K, Abonia JP, Brandt EB, Cohen E, Blanchard C, et al IL-9- and mast cell-mediated intestinal permeability predisposes to oral antigen hypersensitivity. J Exp Med. 2008;205(4):897–913. 10.1084/jem.20071046 . 18378796 PMC2292227

[58] Nei Y, Obata-Ninomiya K, Tsutsui H, Ishiwata K, Miyasaka M, Matsumoto K, et al GATA-1 regulates the generation and function of basophils. Proc Natl Acad Sci U S A. 2013;110(46):18620–5. Epub 2013/10/30. 10.1073/pnas.1311668110 24167252 PMC3831963

[59] Noti M, Kim BS, Siracusa MC, Rak GD, Kubo M, Moghaddam AE, et al Exposure to food allergens through inflamed skin promotes intestinal food allergy through the thymic stromal lymphopoietin-basophil axis. J Allergy Clin Immunol. 2014;133(5):1390–9, 9.e1-6. Epub 2014/02/25. 10.1016/j.jaci.2014.01.021 24560412 PMC4007098

[60] Kweon M-N, Yamamoto M, Kajiki M, Takahashi I, Kiyono H. Systemically derived large intestinal CD4(+) Th2 cells play a central role in STAT6-mediated allergic diarrhea. Journal of Clinical Investigation. 2000;106(2):199–206. PMC314304. 10.1172/JCI8490 10903335 PMC314304

[61] Minai-Fleminger Y, Elishmereni M, Vita F, Rosa Soranzo M, Mankuta D, Zabucchi G, et al Ultrastructural evidence for human mast cell-eosinophil interactions in vitro. Cell and Tissue Research. 2010;341(3):405–15. 10.1007/s00441-010-1010-8 20686785

[62] Elishmereni M, Bachelet I, Nissim Ben-Efraim AH, Mankuta D, Levi-Schaffer F. Interacting mast cells and eosinophils acquire an enhanced activation state in vitro. Allergy. 2013;68(2):171–9. 10.1111/all.12059 23205534

[63] Piliponsky AM, Pickholtz D, Gleich GJ, Levi-Schaffer F. Human eosinophils induce histamine release from antigen-activated rat peritoneal mast cells: a possible role for mast cells in late-phase allergic reactions. J Allergy Clin Immunol. 2001;107(6):993–1000. 10.1067/mai.2001.114656 11398076

